# Nucleotide Excision Repair Protein Rad23 Regulates Cell Virulence Independent of Rad4 in Candida albicans

**DOI:** 10.1128/mSphere.00062-20

**Published:** 2020-02-19

**Authors:** Jia Feng, Shuangyan Yao, Yansong Dong, Jing Hu, Malcolm Whiteway, Jinrong Feng

**Affiliations:** a Department of Pathogen Biology, School of Medicine, Nantong University, Nantong, Jiangsu, China; b Biology Department, Concordia University, Montreal, Quebec, Canada; University of Georgia

**Keywords:** *Candida albicans*, Rad23, Rad4, nucleotide excision repair, virulence

## Abstract

In the pathogenic yeast Candida albicans, the DNA damage response contributes to pathogenicity by regulating cell morphology transitions and maintaining survival in response to DNA damage induced by reactive oxygen species (ROS) in host cells. However, the function of nucleotide excision repair (NER) in C. albicans has not been extensively investigated. To better understand the DNA damage response and its role in virulence, we studied the function of the Rad23 nucleotide excision repair protein in detail. The *RAD23* deletion strain and overexpression strain both exhibit UV sensitivity, confirming the critical role of *RAD23* in the nucleotide excision repair pathway. Genetic interaction assays revealed that the role of *RAD23* in the UV response relies on *RAD4* but is independent of *RAD53*, *MMS22*, and *RAD18*. *RAD4* and *RAD23* have similar roles in regulating cell morphogenesis and biofilm formation; however, only *RAD23*, but not *RAD4*, plays a negative role in virulence regulation in a mouse model. We found that the *RAD23* deletion strain showed decreased survival in a *Candida*-macrophage interaction assay. Transcriptome sequencing (RNA-seq) and quantitative real-time PCR (qRT-PCR) data further revealed that *RAD23*, but not *RAD4*, regulates the transcription of a virulence factor, *SUN41*, suggesting a unique role of *RAD23* in virulence regulation. Taking these observations together, our work reveals that the *RAD23*-related nucleotide excision pathway plays a critical role in the UV response but may not play a direct role in virulence. The virulence-related role of *RAD23* may rely on the regulation of several virulence factors, which may give us further understanding about the linkage between DNA damage repair and virulence regulation in C. albicans.

**IMPORTANCE** Candida albicans remains a significant threat to the lives of immunocompromised people. An understanding of the virulence and infection ability of C. albicans cells in the mammalian host may help with clinical treatment and drug discovery. The DNA damage response pathway is closely related to morphology regulation and virulence, as well as the ability to survive in host cells. In this study, we checked the role of the nucleotide excision repair (NER) pathway, the key repair system that functions to remove a large variety of DNA lesions such as those caused by UV light, but whose function has not been well studied in C. albicans. We found that Rad23, but not Rad4, plays a role in virulence that appears independent of the function of the NER pathway. Our research revealed that the NER pathway represented by Rad4/Rad23 may not play a direct role in virulence but that Rad23 may play a unique role in regulating the transcription of virulence genes that may contribute to the virulence of C. albicans.

## Abstract (precis)

Candida albicans remains a significant threat to the lives of immunocompromised people. An understanding of the virulence and infection ability of C. albicans cells in the mammalian host may help with clinical treatment and drug discovery. The DNA damage response pathway is closely related to morphology regulation and virulence, as well as the ability to survive in host cells. In this study, we checked the role of the nucleotide excision repair (NER) pathway, the key repair system that functions to remove a large variety of DNA lesions such as those caused by UV light, but whose function has not been well studied in C. albicans. We found that Rad23, but not Rad4, plays a role in virulence that appears independent of the function of the NER pathway. Our research revealed that the NER pathway represented by Rad4/Rad23 may not play a direct role in virulence but that Rad23 may play a unique role in regulating the transcription of virulence genes that may contribute to the virulence of C. albicans.

## INTRODUCTION

Candida albicans, whose pathogenicity is related to characteristics such as adhesion to and invasion into host cells, the secretion of hydrolases, the yeast-to-hyphal transition, contact sensing and thigmotropism, and biofilm formation, remains the most prevalent fungal pathogen infecting humans (1). Recently, several studies have established that the DNA damage response is likely to be important for the pathogenesis of C. albicans cells (2–4).

Genome integrity is critical for cells to maintain a normal cell cycle while external and internal factors may lead to DNA damage (5). The accumulation of damaged DNA may disrupt cell cycle progression, which in C. albicans cells may in turn lead to abnormal morphology (6). C. albicans yeast cells treated with either UV stress, the DNA replication inhibitor hydroxyurea (HU), or the DNA methylation agent methyl methane sulfonate (MMS) all exhibit significant filamentous growth (7). Moreover, cells losing appropriate DNA damage repair systems also exhibit abnormal morphogenesis. Deletion of *RAD52*, a gene involved in homologous recombination, causes a strong sensitivity to MMS and also activates filamentous growth (8), and blocking the deactivation of Rad53 by deletion of *PPH3* promotes filamentous growth and causes increased virulence (9–11). Similarly, Rad6 serves a role in protecting against UV damage but also regulates yeast-hypha morphogenesis in C. albicans (12).

The ability to resist DNA damage is also crucial for C. albicans cells to propagate infectively in a host (13). The innate immune system represents the first line of defense to block invading pathogens (14). During this interaction, the reactive oxygen species (ROS) produced by immune cells may damage the genomic DNA of the pathogen, causing events such as chemical base changes, structural alterations, single- and double-strand breaks, and DNA cross-linkage (15, 16). Therefore, proper DNA damage recognition and repair ability are critical for pathogens to maintain virulence. Consistent with this requirement, several DNA damage response or repair proteins in C. albicans, including Rad3, Cad6, Fzd1, and Noc3, have been reported to be highly regulated during the interaction with macrophages (17). Moreover, deletion of the spindle assembly checkpoint component Mad2 in C. albicans causes increased chromosome loss and reduces cell virulence and the ability to survive in macrophages, supporting the relationship between the DNA damage response and pathogenesis (18). Similarly, the histone H3 acetyltransferase Rtt109 plays a critical role in maintaining genome stability, and its deletion results in fungal cells that are significantly less pathogenic in mice and more susceptible to killing by macrophages *in vitro* than are wild-type cells (19).

Given the critical role in pathogenicity, the DNA damage signal pathways in C. albicans have been widely studied. But compared with Rad52-related DNA damage repair and Rad53-related checkpoint pathways, the nuclear excision repair (NER) system remains underinvestigated. In both prokaryotes and eukaryotes, nuclear excision repair represents the key repair system that functions to remove a large variety of DNA lesions induced by treatments such as UV light (20, 21). In this study, we focus on a nucleotide excision protein, Rad23, whose ortholog in Saccharomyces cerevisiae plays dual roles in nucleotide excision as well as ubiquitin-mediated protein degradation (22, 23). Here, we establish that Rad23 plays a critical, Rad4-dependent role in the UV-induced DNA damage response. Nevertheless, Rad23, but not Rad4, plays a positive role in virulence through its regulation of a virulence factor, *SUN41*.

## RESULTS

### Sequence analysis of *RAD23* in C. albicans.

**FIG 1:**
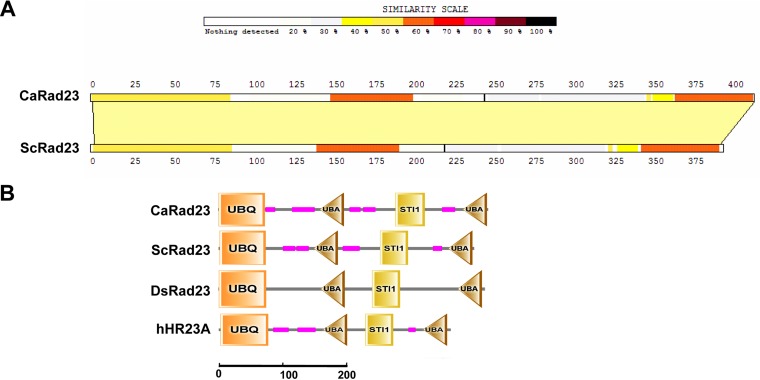
Sequence alignment of CaRad23 and ScRad23. (A) The sequences of CaRad23 and ScRad23 were aligned by the SIM online tool (https://web.expasy.org/sim/). (B) Comparison of protein secondary structure of CaRad23 to the potential orthologs of Saccharomyces cerevisiae, Drosophila simulans (DsRad23), and Homo sapiens (hHR23A). UBQ, ubiquitin domain; UBA, ubiquitin-associated domain; STI1, heat shock chaperonin-binding motif.

S. cerevisiae Rad23 (ScRad23) is a key component of nucleotide excision repair factor 2 (NEF2), and deletion of *ScRAD23* renders cells sensitive to UV-induced DNA damage (24). From the C. albicans genomic database (www.candidagenome.org), we obtained the sequence of the candidate C. albicans Rad23 (CaRad23), encoding a protein of 417 amino acids (aa) that shares ∼41% sequence identity with ScRad23 (Fig. 1A). The CaRad23 protein contains four internal domains, including a ubiquitin (UBQ) domain (aa 1 to 72), two ubiquitin-associated (UBA) domains (aa 157 to 194 and aa 378 to 415), and an STI1 domain (aa 274 to 319), which are conserved in Rad23 proteins from the model fungus S. cerevisiae and other organisms.

### Deletion of *RAD23* creates defects in cytokinesis and nuclear segregation and enhances genome instability.

**FIG 2:**
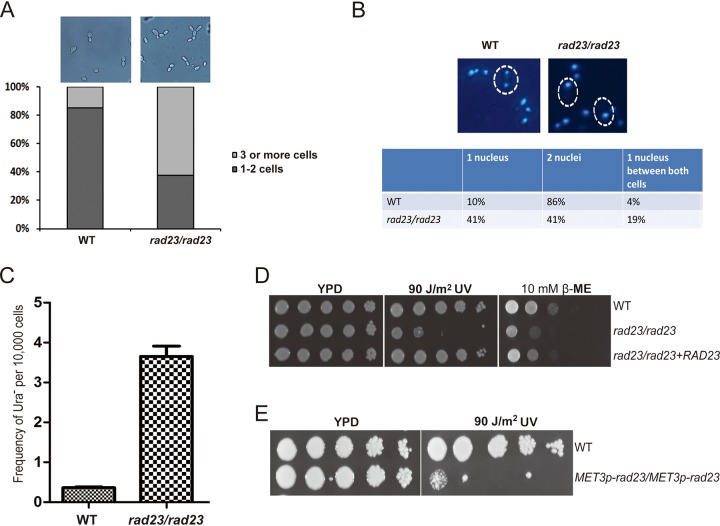
Deletion of *RAD23* generates defects in cell division and sensitivity to UV light. (A) Both the wild type (WT) and the *RAD23* deletion strain (*rad23/rad23*) were grown in liquid YPD medium. Cell morphologies are shown. Percentages of cells in chains were determined by dividing the sum of cells in chains by the total number of cells. Over 200 cells were counted for each group. (B) Both the wild type and the *RAD23* deletion strain were stained with DAPI. The cells with buds containing different types of nuclei were divided into three groups. (C) The frequency of losing the heterozygous *URA3* marker was assessed by comparing the colony count on YNB medium plus 5-FOA with that on normal YPD plates. The experiment was replicated three times. (D) Phenotype assay of the *RAD23* deletion strain in response to genotoxic stress. (E) Phenotype assay of the *RAD23* overexpression strain in response to UV light. The indicated strains were grown overnight in YPD medium at 30°C, serially diluted, and spotted onto YPD plates with or without the indicated treatments. Plates were incubated at 30°C for 2 to 3 days.

To further check the function of *RAD23* in C. albicans, we deleted *RAD23* in the SN148 strain background. Cells with a deletion of *RAD23* displayed normal morphology but showed an increased number of attached cells. Quantification of cell chains with three or more cells revealed that 61% of *RAD23* deletion cells are in chains whereas only 5% are in chains in wild-type cells (Fig. 2A).

Because a potential DNA damage repair role may be related to nuclear segregation, we checked nuclear morphogenesis through 4′,6′-diamidino-2-phenylindole (DAPI) staining (Fig. 2B). In the case of the wild-type strain, 86% (*n* > 50) of large budded cells had two nuclei, one in each bud and the mother cell. In contrast, in *RAD23* deletion cells, only 41% (*n* > 50) of large budded cells had two nuclei, and another 41% of large budded cells had one nucleus in the mother cell, indicating a possible defect in nuclear segregation.

To test the function of Rad23 in regulating genomic stability, we constructed a heterozygous *URA3* strain to investigate the loss of heterozygosity through a 5-fluoroorotic acid (5-FOA) resistance assay. We found that the wild-type strain had a low frequency of cells lacking the *URA3* marker (0.37 × 10^−4^), while the *RAD23* deletion strain had roughly a 10-fold-higher frequency of loss of this marker (3.77 × 10^−4^), indicating that Rad23 plays a role in maintaining genome stability (Fig. 2C).

### Both deletion and overexpression of *RAD23* cause sensitivity to UV stress.

Deletion of *RAD23* rendered C. albicans cells sensitive to UV, and the introduction of *RAD23* back into the homozygous mutant rescued the sensitive phenotype, supporting a role of *RAD23* in the nuclear excision repair pathway (Fig. 2D). In contrast, we found that the deletion of *RAD23* had no significant impact on the response to MMS, HU, SDS, or Congo red. Here, we also observed that deletion of *RAD23* resulted in sensitivity to β-mercaptoethanol (β-ME), suggesting a potential role in the unfolded protein response (25).

To further check the function of Rad23 in the UV response, we constructed a *RAD23* overexpression strain using an *MET3* promoter and found that overexpression of *RAD23* also resulted in sensitivity to UV stress (Fig. 2E), further suggesting an important role for Rad23 in the UV response.

### Genetic interaction analysis of *RAD23* in response to UV-induced DNA damage.

In S. cerevisiae, Rad23 and Rad4 were reported to form a complex, nuclear excision repair factor 2 (NEF2), that binds to the damaged DNA (26, 27) and recruits Rad2, Rad10, and Rad1 to correct the damage. In order to explore the detailed role of *RAD23* in the response to DNA damage in C. albicans, we performed a genetic interaction analysis.

We investigated double-mutant strains involving *RAD23* and various genes which have potential roles in the UV response. Rad53, the main checkpoint kinase in C. albicans (7), was chosen to address a potential relationship of Rad23 with DNA damage signal transduction. Rad4 was chosen to represent the NER group in which Rad4 is part of the NEF2 complex. In addition, we combined the *RAD23* null mutation with Pph3, a catalytic subunit of protein phosphate 4 (PP4) that functions to control the deactivation of Rad53 during the recovery from or adaption to DNA damage (9, 11). Rad18, a representative of the Rad6 epistasis group, was chosen as a component of postreplication repair (28). Mms22, a putative adaptor subunit of an E3 ubiquitin ligase complex, was chosen to represent the replication repair pathway (29). Hof1, whose deletion strain shows sensitivity to genotoxic stress, including UV stress, was chosen to address the potential relationship between cytokinesis and nuclear excision repair.

**FIG 3:**
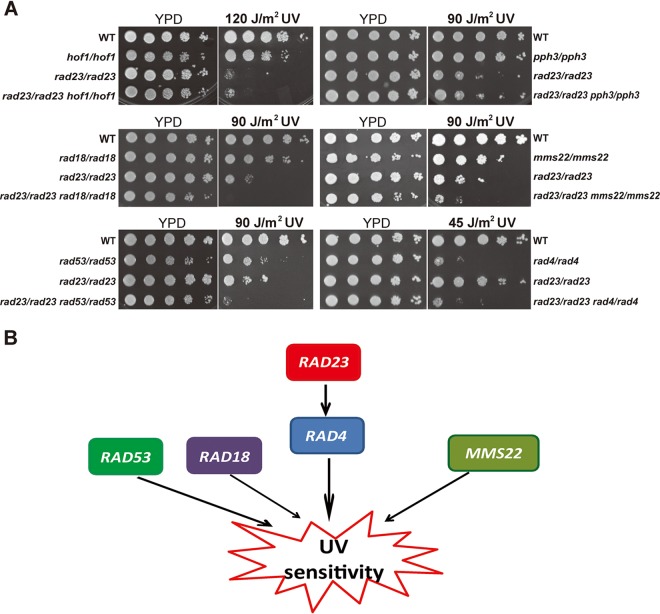
Genetic epistasis analysis of *RAD23.* (A) Increased UV sensitivity was found in a strain with the double deletion of *RAD23* with *MMS22*, *RAD18*, or *RAD53*, but not in a strain with the double deletion of *RAD23* with *HOF1*, *PPH3*, or *RAD4*. (B) A genetic interaction relationship between *RAD23* and other DNA damage-related genes was shown.

Deletion of either *RAD18*, *RAD53*, or *MMS22* resulted in a UV-sensitive phenotype, and the sensitivity to UV of double mutants of *RAD23* with *RAD18*, *RAD53*, or *MMS22* was increased relative to that of the single mutants (Fig. 3A), suggesting a role of *RAD23* independent from these genes in the UV response. In addition, a strain with a deletion of *HOF1* (46) or *PPH3* (9) was reported to show a slight sensitivity to UV, and a strain with the double deletion of *RAD23* and *HOF1* or *PPH3* showed no typically increased sensitivity to UV, suggesting that *PPH3* or *HOF1* plays a less important role in the UV response than *RAD23*. Furthermore, deletion of *RAD4* and *RAD23* caused sensitivity to UV; however, the *RAD4 RAD23* double deletion mutant showed UV sensitivity similar to that of the *RAD4* single-deletion strain, consistent with *RAD4* and *RAD23* functioning in the same pathway in response to UV (Fig. 3A and B).

### *RAD23* and *RAD4* play negative roles in biofilm formation.

**FIG 4:**
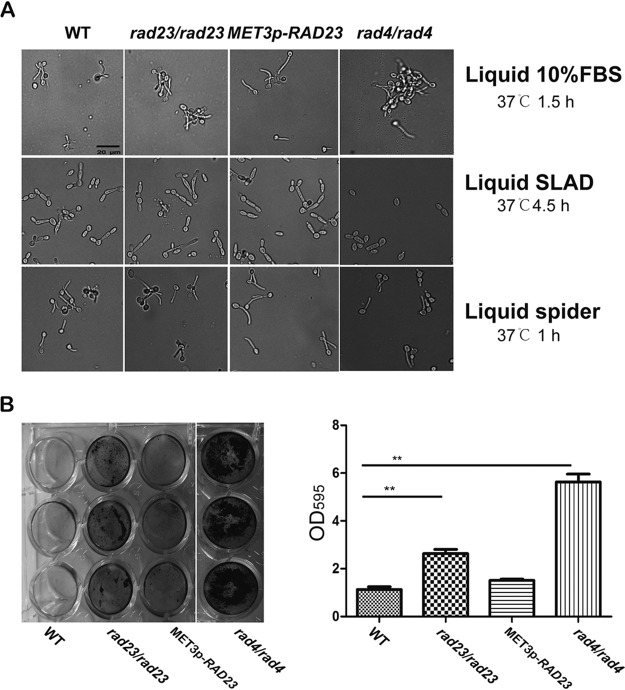
*RAD23* and *RAD4* regulate biofilm formation. (A) Filamentation of *Candida* cells in liquid Spider and SLAD media and in YPD medium containing 10% FBS. Four strains were grown to the stationary phase in liquid YPD medium overnight at 30°C, diluted 10 times, and grown at 37°C in liquid Spider medium for 1 h, in SLAD medium for 4.5 h, and in YPD medium containing 10% FBS medium for 1.5 h to induce hypha formation before photos were taken. (B) Biofilm formation ability of the *RAD23* or *RAD4* mutant. Four strains were grown to the stationary phase in liquid YNB medium at 30°C, diluted to an OD_600_ of 1, transferred to a 24-well plate, and kept at 37°C for 48 h. Cells were washed with PBS and stained with crystal violet. Plates were scanned (left panel) after removal of staining solution and four washes of individual plate wells with sterile distilled water. The absorbance of destaining solution at an OD_595_ was measured and averaged. Error bars indicate standard deviations. **, *P* < 0.01 (two-tailed *t* test).

DNA damage proteins are involved in regulating cell morphology and virulence. Here, we tested roles in hyphal formation in liquid yeast extract-peptone-dextrose (YPD) medium containing fetal bovine serum (FBS), in Spider medium, and in solid synthetic low-ammonium–dextrose (SLAD) medium by deleting *RAD23* and *RAD4*. In these hyphal-inducing media, the *RAD23* deletion cells, *RAD23* overexpression cells, and *RAD4* deletion cells formed hyphae normally, similar to the wild-type cells (Fig. 4A). On solid YPD medium with 10% FBS, colonies of both the *RAD23* deletion cells and *RAD23* overexpression strains showed rough, crenulated surfaces similar to those generated by the wild-type cells (data not shown).

Filamentous growth is directly associated with biofilm formation, which is a critical virulence factor of C. albicans cells (30). Here, we tested the biofilm formation of C. albicans cells in strains with deletions of *RAD23* and *RAD4*. As shown in Fig. 4B, deletion of *RAD23* increased biofilm formation compared with the level in wild-type cells while overexpression of *RAD23* generated biofilm formation at a level similar to that of wild-type cells. As with the *RAD23* deletion cells, deletion of *RAD4* also increased the level of biofilm formation.

### *RAD23*, but not *RAD4*, influences cellular virulence in a mouse model.

**FIG 5:**
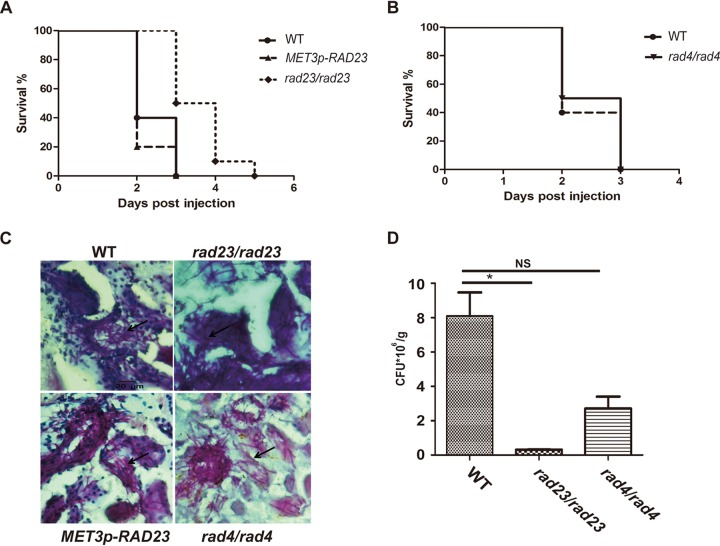
*RAD23* deletion influences cellular virulence in a mouse model. (A and B) Survival curves of mice intravenously infected with the C. albicans strains indicated. BALB/c male mice (5 weeks old; 10 mice in each group) were injected with 2 × 10^6^ stationary-phase cells. Mice were checked every day for morbidity. Survival was monitored for 5 days. (C) For histopathological examination, kidney tissues of moribund mice were obtained from mice at day 2 after infection. The infected kidney tissues were stained with periodic acid-Schiff reagent. The hyphae are indicated by arrows. (D) For fungal burden examination, kidney tissues were obtained from 2 mice from each group after infection for 48 h. The left kidney was ground and spread on YPD medium with dilutions. The average data of 2 mice are shown. *, *P* < 0.05; NS, not significant (unpaired two-tailed *t* test).

To investigate the role of Rad23 in virulence, a mouse infection model was assayed. All mice infected with 2 × 10^6^ wild-type cells died on day 3 after inoculation, with an average life span of 2 days per mouse, while all mice infected with the *RAD23* deletion cells died on day 5 after inoculation, with an average life span of 3.5 days per mouse (Fig. 5A). In contrast, the mice infected with the *RAD23* overexpression strain had a life span of 2 days, similar to that of mice inoculated with the wild-type cells.

Since Rad23 and Rad4 play correlated roles in response to DNA damage, we also checked the role of Rad4 in virulence. Strikingly, the mice infected with *RAD4* deletion cells showed an average life span of 2 days per mouse, similar to that of mice inoculated with the wild-type strain (Fig. 5B).

Histological examination revealed that the *RAD23* deletion cells, *RAD23* overexpression cells, and *RAD4* deletion cells mainly existed in hyphal forms in infected mouse kidney tissues, similar to findings in the wild-type cells (Fig. 5C).

To assess the survival ability of *RAD23* and *RAD4* deletion cells in the host, the kidneys from infected mice were taken out after 48 h of infection and ground for CFU assays. We found 8.10 × 10^6^ CFU/g with infection with the wild-type strain, 2.70 × 10^6^ CFU/g with the *RAD4* deletion strain, and only 0.32 × 10^6^ CFU/g with the *RAD23* deletion strain (Fig. 5D).

### *RAD23* regulates the survival of C. albicans cells in macrophages.

**FIG 6:**
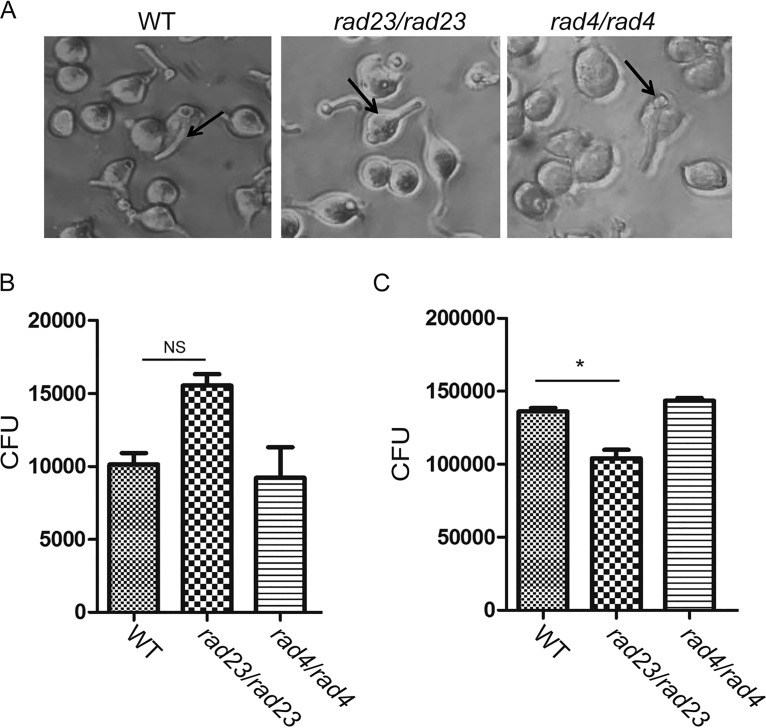
*Candida* cell-macrophage interaction assay. *Candida* cells were coincubated with macrophages at an MOI of 1:1. After 2 h of incubation, the plates were washed with PBS three times before being imaged (A). The *Candida* cells in one plate were released by 0.01% SDS and spread on YPD plates with dilutions. The remaining plate was kept for another 6 h. The cells were then released using the same protocol. The numbers of *Candida* cells after incubation for 2 h (B) or 8 h (C) are shown. * *P* < 0.05; NS, not significant (two-tailed *t* test).

Since deletion of *RAD23* has no dramatic impact on hyphal formation but does impair virulence in the mouse model, we investigated whether Rad23 may regulate cell survival in host immune cells. We performed a macrophage (RAW264.7)-*Candida* cell interaction assay. After coincubation for 2 h, *RAD23* and *RAD4* deletion cells were engulfed similarly to wild-type cells into macrophages; 1.55 × 10^4^ CFU/well were found using macrophages infected with *RAD23* deletion cells while 1.01 × 10^4^ CFU/well were found with wild-type cells, and 0.92 × 10^4^ CFU/well were found with *RAD4* deletion cells (Fig. 6). However, after further incubation for 6 h, 1.04 × 10^5^ CFU/well were found in macrophages infected with *RAD23* deletion cells, which is significantly less than the level with wild-type cells (1.36 × 10^5^ CFU/well). In contrast, 1.43 × 10^5^ CFU/well were found using *RAD4* deletion cells (Fig. 6).

### Rad23 plays a negative role in the regulation of virulence-related genes.

**FIG 7:**
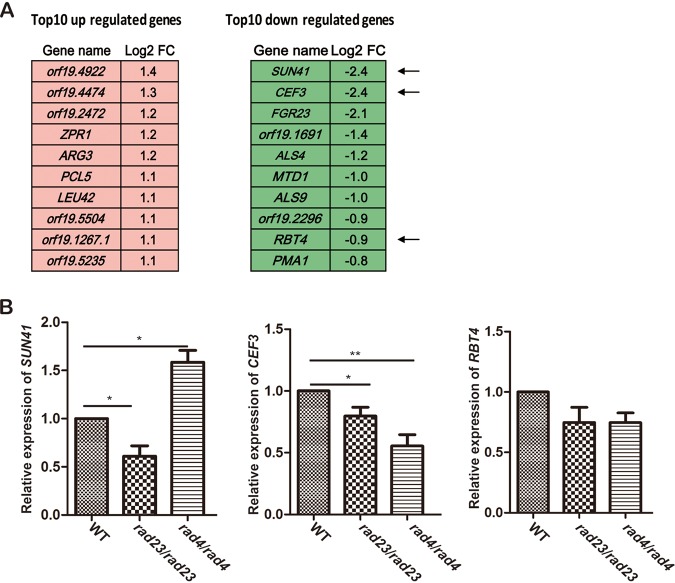
Gene expression impact of deletion of *RAD23*. (A) Top 10 genes whose transcription levels are significantly increased or decreased, as indicated, in a *RAD23* deletion strain with MMS treatment for 2 h. (B) Real-time PCR analysis of the expression levels of virulence-related genes after deletion of *RAD23* and *RAD4*. The cells were cultured in liquid YPD medium overnight and then diluted in fresh YPD medium for 3 h before being harvested for RNA extraction. The results shown here are average data of three independent experiments. **, *P* < 0.01; *, *P* < 0.05 (two-tailed *t* test).

To better understand the impact of *RAD23* on control of virulence, a transcriptome sequencing (RNA-seq) assay was performed to check what genes are affected by deleting *RAD23*. Since host cells may produce ROS to damage genomic DNA, we used MMS to treat cells to mimic a DNA damage environment. Using statistical-significance analysis with a false-discovery rate (FDR) value of less than 0.01 and a cutoff of 0.5-fold, we identified 87 genes whose levels of expression were significantly affected by *RAD23* deletion; 46 genes were upregulated, and 41 were downregulated (Table S3). Among upregulated genes, *CUZ1* and *BUL1* are related to the ubiquitin/proteasome-mediated protein degradation pathway, which supports a role of *RAD23* in the ubiquitin/proteasome pathway (Fig. 7A). Among the downregulated group, several genes, including *SUN41*, *CEF3*, and *RBT4*, have been reported to play critical roles in regulating the virulence of C. albicans cells (31–34), suggesting that regulation by Rad23 is a mechanism for maintaining cell virulence.

We used quantitative real-time PCR (qRT-PCR) to confirm the downregulation of these virulence-related genes, which showed a result consistent with the RNA-seq data. *CEF3* was found to be downregulated in both *RAD23* and *RAD4* deletion cells, while *SUN41* was found to be downregulated only in *RAD23* deletion cells and not in *RAD4* deletion cells (Fig. 7B), suggesting a possible unique Rad23 function in regulating *SUN41*.

## DISCUSSION

In this study, we characterized the function of a potential nuclear excision protein, Rad23, in the DNA damage response and virulence in C. albicans. Deletion of *RAD23* renders cells sensitive to UV stress and increases genome instability. The function of Rad23 in the UV response relies on another nuclear excision protein, Rad4. However, deletion of *RAD23* but not *RAD4* attenuates virulence and the survival ability in macrophages, perhaps indicating the role of *RAD23* in regulating the virulence factor *SUN41*.

In S. cerevisiae, Rad23 together with Rad4 forms the NEF2 complex that binds damaged DNA and recruits repair proteins to correct UV-induced damage (35). In this study, we confirmed that Rad23 of C. albicans appears to play a similar role. Genetic interaction assays showed that the function of Rad23 in the UV response relies on Rad4. Given that Rad23 in S. cerevisiae has been reported to maintain the stability of Rad4 (23), we propose that Rad4 plays a critical role in binding damaged DNA, while Rad23 plays an indirect role in the NER pathway through stabilizing Rad4, which may explain why Rad4 plays a dominant role in the UV response.

In addition, we found that the role of Rad23 in the UV response is independent of Rad53, the main checkpoint kinase. In S. cerevisiae, Rad53 has been reported to be a critical checkpoint kinase to receive the DNA damage signal and stop the cell cycle to activate the expression of repair genes to correct damaged DNA. Our genetic interaction data indicate that Rad23 is not involved in the *RAD53*-related pathway. But, strikingly, UV sensitivity caused by deleting *PPH3*, which encodes a catalytic subunit of Rad53, is not increased by further deleting *RAD23*, suggesting a potential correlative pathway of *PPH3* and *RAD23* in the UV response. Since Pph3 may be involved with other DNA damage repair components, like Rfa2 (36), there should be other checkpoint kinases/damage repair proteins involved in the Rad23/Rad4-related UV response pathway.

DNA damage could be related to cell virulence of C. albicans during infection in the host. Through checking cell virulence in a mouse model, we found that Rad23, independent of Rad4, plays a role in maintaining cell virulence. Since Rad23 interacts with Rad4 *in vivo*, the different levels of cell virulence could represent an independent role of Rad23 itself. Recently, a study in Cryptococcus neoformans revealed that *RAD23* regulates cell virulence independent of its role in nucleotide excision DNA repair, which may support our finding in C. albicans (37). Consistent with this, overexpression of *RAD23* resulted in increased sensitivity to UV but had no impact on virulence, further supporting the idea that cell virulence regulation by Rad23 may be separate from its role in nuclear damage excision.

The pathogenesis of C. albicans cells relies on several factors, such as morphogenesis transition, biofilm formation, and secreted proteases. Here, no difference of levels of hyphal formation was found by deleting *RAD23* and *RAD4*, suggesting that other factors may contribute to the changes in virulence. Through RNA-seq and qRT-PCR, we found that both *RAD23* and *RAD4* play critical roles in maintaining the expression levels of several virulence factor genes. As the decreased expression of *SUN41* was found only in *RAD23* deletion cells, the key target of *RAD23* in regulating virulence may be *SUN41*. In C. albicans, *SUN41* encodes a secreted protein, and its deletion causes increased cell virulence and decreased biofilm formation (31, 38, 39). So, the low expression of *SUN41* may explain the decreased cell virulence caused by deleting *RAD23* in the mouse model. Nevertheless, the increased biofilm formation in *RAD4* and *RAD23* deletion cells may suggest that Sun41 plays a secondary role in biofilm formation. The increased expression of other genes may contribute to the increased biofilm formation.

In general, we propose a regulatory model of Rad23 in the UV response and virulence. Rad4 plays a critical role in the UV response, while Rad23 may support the function of Rad4, and probably its stability, in the UV response. The roles of Rad53, Mms22, and Rad18 in the UV response do not rely on Rad23/Rad4. On the other hand, only Rad23, but not Rad4, plays a critical role in virulence regulation, which may rely on the regulation of *SUN41* expression. Further work will be needed to test these ideas.

## MATERIALS AND METHODS

### Strains, media, and reagents.

C. albicans strains were grown in YPD medium with 50 mg of uridine per liter as described previously (40). Strains and primers used in this study are listed in Table S1 and Table S2, respectively. Methyl methane sulfonate (MMS) was purchased from Sigma-Aldrich (USA). Hydroxyurea (HU) and other chemicals were purchased from Sangon (China). The yeast nitrogen base (YNB) medium was supplemented with appropriate nutrients for plasmid selection and maintenance. The *MET3* promoter-induced *RAD23* overexpression strain was cultured in YNB medium without Cys and Met. To induce hypha formation, Spider and SLAD medium were prepared as described previously (41). Solid medium contained 2% agar.

10.1128/mSphere.00062-20.1 TABLE S1 Strains used in this study. Download Table S1, DOCX file, 0.01 MB. Copyright © 2020 Feng et al. 2020 Feng et al. This content is distributed under the terms of the Creative Commons Attribution 4.0 International license.

10.1128/mSphere.00062-20.2 TABLE S2 Primers used in this study. Download Table S2, DOCX file, 0.01 MB. Copyright © 2020 Feng et al. 2020 Feng et al. This content is distributed under the terms of the Creative Commons Attribution 4.0 International license.

10.1128/mSphere.00062-20.3 TABLE S3 Genes whose transcription was affected by deleting *RAD23*. Download Table S3, DOCX file, 0.02 MB. Copyright © 2020 Feng et al. 2020 Feng et al. This content is distributed under the terms of the Creative Commons Attribution 4.0 International license.

### Gene deletion, rescue, and epitope tagging of proteins.

To construct a *RAD23* deletion strain in C. albicans, we used a transient CRISPR/Cas9 system (42). In general, the Cas9 gene was amplified with common primers P7 and P8. The single guide RNA (sgRNA) was amplified by annealing PCR with primers P5 and P6, using the products of two separate PCRs containing the sgRNA sequence, while repair DNA was amplified from pFA-HIS1. Finally, the Cas9, sgRNA, and repair DNA products were transformed into the wild-type strain and selected on synthetic dextrose (SD) plates lacking His. The correct knockout strains were confirmed by PCR. Similarly, an *RAD4* deletion strain was constructed by a transient CRISPR/Cas9 system.

To knock out *RAD23* in the *MMS22*, *RAD4*, *PPH3*, *RAD18*, *HOF1*, or *RAD53* deletion strain, a similar transient CRISPR/Cas9 protocol was used.

To rescue the phenotype of the *RAD23* deletion strain, a 3,031-bp DNA fragment containing the full-length *RAD23* gene was amplified with primers RAD23-F and RAD23-R and cloned into the KpnI site of plasmid CIP10 (43), generating pCIP10*-RAD23*. Then, pCIP10-*RAD23* was linearized by StuI and integrated at the RP10 locus of the genome. The integration was confirmed using primers URA-TF and RPS-F.

To construct the *RAD23* overexpression strain, an *MET3* promoter was amplified from pFA-URA3-MTE3 to replace two copies of the original promoters of the *RAD23* gene by a transient CRISPR/Cas9 system.

### Genome stability assay.

To assess genome stability (44), the CIP10 plasmid (43) containing a *URA3* marker was linearized by StuI and integrated into the RP10 locus of both the wild-type strain and the *RAD23* deletion strain, generating heterozygous *URA3*^+^ strains. The strains were streaked on YNB-dextrose-Ura medium twice and inoculated in YPD medium overnight at 30°C. The next day, cells were harvested and washed twice with distilled water. For each strain, 100 μl of 10^−1^ cells was spotted on YNB dextrose 5-FOA plates while 20 μl of 10^−4^ cells was spotted on YPD plates to check the cell numbers. All the strains were grown as duplicates in three independent samples. Finally, the average numbers of cells on YNB–dextrose–5-FOA plates and YPD plates were calculated.

### RNA extraction, RNA-seq assay, and qRT-PCR.

Single colonies of the C. albicans wild-type strain SN148 and the *RAD23* deletion strain were each inoculated into 3 ml of YPD medium and incubated overnight at 30°C on a 220-rpm shaker. The overnight cultures of two independent colonies were diluted to an optical density at 600 nm (OD_600_) of 0.2 in 10 ml of YPD medium and grown to an OD_600_ of 0.6 at 30°C with shaking at 220 rpm. The cells were treated with 0.02% MMS for 2 h before being harvested for RNA extraction.

The RNA extraction was performed by Beijing Biomics Biotech Co., Ltd. RNA purity was checked using a NanoPhotometer spectrophotometer (Implen, CA), and RNA integrity was assessed using an Agilent 2100 Bioanalyzer system with an RNA Nano 6000 kit (Agilent Technologies, CA). Sequencing libraries were generated using an NEB Next Ultra RNA Library Prep kit for Illumina (NEB, USA) according to the manufacturer’s recommendations, and index codes were added to assign sequences to each sample. The clustering of the index-coded samples was performed on a cBot Cluster Generation System using a TruSeq PE (paired-end) Cluster kit v3-cBot-HS (Illumina, USA) according to the manufacturer’s instructions. After cluster generation, the library preparations were sequenced on an Illumina Hiseq2500/X platform and 125/150-bp paired-end reads were generated. C. albicans SC5314, version A22 downloaded from the *Candida* Genome Database (CGD) (http://www.candidagenome.org/), was used as a reference genome.

For quantitative real-time PCR (qRT-PCR) analysis, a Synthesis SuperMix (TransGen, China) containing a genomic DNA (gDNA) remover was employed for cDNA synthesis, and a SYBR Premix Ex Taq RT-PCR kit (TaKaRa, Japan) was used on an Eco Realtime PCR system (Illumina, USA).

### Filamentation test.

Filamentation in liquid medium was assessed by inoculating cells with an appropriate dilution into the YPD medium plus 10% fetal bovine serum (FBS), Spider medium, or SLAD medium and growing cultures with shaking at 37°C (41). The cells were imaged on a Leica DM5000B microscope with a 40× objective.

Filamentation on solid medium was also examined on YPD plates containing 10% FBS. About 20 cells of each strain was spread onto one plate, and plates were incubated for 5 to 7 days at 37°C before imaging.

### Biofilm assay.

Biofilm assays were performed as previously described (45). Single colonies of the experimental strains were inoculated into 3 ml of YNB-dextrose medium and grown overnight at 30°C. Each overnight culture was diluted to an OD_600_ of 1.0, added into a 24-well polystyrene plate (NEST, China), and kept for 48 h at 37°C. The YNB medium was then decanted, and the remaining cells were washed three times with 700 μl of 0.01 M phosphate-buffered saline (PBS). Samples were allowed to air dry for 45 min before being stained with 385 μl of 0.4% (wt/vol) aqueous crystal violet for a further 45 min. Wells were washed four times with 1 ml of sterile distilled water. Each culture was then destained by addition of 700 μl of 95% ethanol. After samples were kept at room temperature for 45 min, 200 μl of each destaining solution was transferred to a 96-well plate, and then 5-fold dilutions were performed in 95% ethanol (final volume of 200 μl); the absorbance at OD_595_ was measured and averaged. The experiment was run in triplicate for each strain.

### Virulence studies.

Male BALB/c mice 5 weeks of age were used for *in vivo* virulence studies as described previously (11). Briefly, 200 μl of 1 × 10^7^ ml^−1^ cells was injected intravenously into the tail vein of each mouse. Survival curves were generated according to the Kaplan-Meier method using the Prism program (GraphPad Software) and compared using a log rank test. Sections were prepared from the kidneys of moribund mice and stained with periodic acid-Schiff (PAS) stain for histological examination (11). The kidney from infected mouse was taken after infection for 48 h and then ground for fungal burden assay. Mouse studies were carried out under the guidelines established by the Ethics Committee of Nantong University, China.

### Survival ability assay of C. albicans cells in macrophages.

RAW264.7 mouse macrophages (5 × 10^4^) were inoculated into 12-well plates and challenged with C. albicans at a multiplicity of infection (MOI) of 1:1. After 2 h of coincubation at 37°C with 5% CO_2_, nonphagocytosed fungal cells were removed by washing plates with phosphate-buffered saline (PBS). To check the engulfed cells, one of the plates was treated with 500 μl of 0.01% SDS in each well, and released cells were spread on YPD plates with 10-fold dilutions. To show the engulfment by macrophages, the CFU were counted, and results were compared with the original cell numbers before coincubation. For the survival assay, the plates were kept for another 6 h at 37°C with 5% CO_2_ after removal of nonphagocytosed fungal cells. The fungal cells inside macrophages were released and counted as before.

### Data availability.

Raw and processed data have been submitted to the NCBI under accession numbers SAMN13781835, SAMN13781836, SAMN13781837, and SAMN13781838.

## References

[1] Whiteway M, Bachewich C 2007 Morphogenesis in Candida albicans. Annu Rev Microbiol 61:529–553. doi:10.1146/annurev.micro.61.080706.093341. 17506678 PMC4452225

[2] da Rosa JL, Kaufman PD 2012 Chromatin-mediated Candida albicans virulence. Biochim Biophys Acta 1819:349–355. doi:10.1016/j.bbagrm.2011.08.007. 21888998 PMC3243783

[3] Hao B, Clancy CJ, Cheng S, Raman SB, Iczkowski KA, Nguyen MH 2009 Candida albicans RFX2 encodes a DNA binding protein involved in DNA damage responses, morphogenesis, and virulence. Eukaryot Cell 8:627–639. doi:10.1128/EC.00246-08. 19252121 PMC2669197

[4] Feng J, Shan A, Hu J, Cao Z, Lv R, Feng J 2019 Genetic interaction between Ptc2 and protein phosphatase 4 (PP4) in the regulation of DNA damage response and virulence in Candida albicans. FEMS Yeast Res 19:foz075. doi:10.1093/femsyr/foz075. 31644792

[5] Pan X, Ye P, Yuan DS, Wang X, Bader JS, Boeke JD 2006 A DNA integrity network in the yeast Saccharomyces cerevisiae. Cell 124:1069–1081. doi:10.1016/j.cell.2005.12.036. 16487579

[6] Loll-Krippleber R, d'Enfert C, Feri A, Diogo D, Perin A, Marcet-Houben M, Bougnoux M-E, Legrand M 2014 A study of the DNA damage checkpoint in Candida albicans: uncoupling of the functions of Rad53 in DNA repair, cell cycle regulation and genotoxic stress-induced polarized growth. Mol Microbiol 91:452–471. doi:10.1111/mmi.12471. 24286230

[7] Shi QM, Wang YM, Zheng XD, Lee RT, Wang Y 2007 Critical role of DNA checkpoints in mediating genotoxic-stress-induced filamentous growth in Candida albicans. Mol Biol Cell 18:815–826. doi:10.1091/mbc.e06-05-0442. 17182857 PMC1805102

[8] Andaluz E, Ciudad T, Gomez-Raja J, Calderone R, Larriba G 2006 Rad52 depletion in Candida albicans triggers both the DNA-damage checkpoint and filamentation accompanied by but independent of expression of hypha-specific genes. Mol Microbiol 59:1452–1472. doi:10.1111/j.1365-2958.2005.05038.x. 16468988

[9] Sun LL, Li WJ, Wang HT, Chen J, Deng P, Wang Y, Sang JL 2011 Protein phosphatase Pph3 and its regulatory subunit Psy2 regulate Rad53 dephosphorylation and cell morphogenesis during recovery from DNA damage in Candida albicans. Eukaryot Cell 10:1565–1573. doi:10.1128/EC.05042-11. 21890819 PMC3209060

[10] Feng J, Zhao Y, Duan Y, Jiang L 2013 Genetic interactions between protein phosphatases CaPtc2p and CaPph3p in response to genotoxins and rapamycin in Candida albicans. FEMS Yeast Res 13:85–96. doi:10.1111/1567-1364.12012. 23083206

[11] Feng J, Duan Y, Qin Y, Sun W, Zhuang Z, Zhu D, Jiang L 2017 The N-terminal pY33XL motif of CaPsy2 is critical for the function of protein phosphatase 4 in CaRad53 deactivation, DNA damage-induced filamentation and virulence in Candida albicans. Int J Med Microbiol 307:471–480. doi:10.1016/j.ijmm.2017.09.017. 28967545

[12] Leng P, Sudbery PE, Brown AJ 2000 Rad6p represses yeast-hypha morphogenesis in the human fungal pathogen Candida albicans. Mol Microbiol 35:1264–1275. doi:10.1046/j.1365-2958.2000.01801.x. 10712706

[13] Uwamahoro N, Verma-Gaur J, Shen HH, Qu Y, Lewis R, Lu JX, Bambery K, Masters SL, Vince JE, Naderer T, Traven A 2014 The pathogen Candida albicans hijacks pyroptosis for escape from macrophages. mBio 5:e00003-14. doi:10.1128/mBio.00003-14. 24667705 PMC3977349

[14] Netea MG, Brown GD, Kullberg BJ, Gow NA 2008 An integrated model of the recognition of Candida albicans by the innate immune system. Nat Rev Microbiol 6:67–78. doi:10.1038/nrmicro1815. 18079743

[15] Fang FC 2011 Antimicrobial actions of reactive oxygen species. mBio 2:e00141-11. doi:10.1128/mBio.00141-11. 21896680 PMC3171981

[16] Dantas A. d S, Day A, Ikeh M, Kos I, Achan B, Quinn J 2015 Oxidative stress responses in the human fungal pathogen, Candida albicans. Biomolecules 5:142–165. doi:10.3390/biom5010142. 25723552 PMC4384116

[17] O’Meara TR, Duah K, Guo CX, Maxson ME, Gaudet RG, Koselny K, Wellington M, Powers ME, MacAlpine J, O’Meara MJ, Veri AO, Grinstein S, Noble SM, Krysan D, Gray-Owen SD, Cowen LE 2018 High-throughput screening identifies genes required for Candida albicans induction of macrophage pyroptosis. mBio 9:e01581-18. doi:10.1128/mBio.01581-18. 30131363 PMC6106084

[18] Bai C, Ramanan N, Wang YM, Wang Y 2002 Spindle assembly checkpoint component CaMad2p is indispensable for Candida albicans survival and virulence in mice. Mol Microbiol 45:31–44. doi:10.1046/j.1365-2958.2002.02995.x. 12100546

[19] Lopes da Rosa J, Boyartchuk VL, Zhu LJ, Kaufman PD 2010 Histone acetyltransferase Rtt109 is required for Candida albicans pathogenesis. Proc Natl Acad Sci U S A 107:1594–1599. doi:10.1073/pnas.0912427107. 20080646 PMC2824404

[20] Waters R, Evans K, Bennett M, Yu SR, Reed S 2012 Nucleotide excision repair in cellular chromatin: studies with yeast from nucleotide to gene to genome. Int J Mol Sci 13:11141–11164. doi:10.3390/ijms130911141. 23109843 PMC3472735

[21] Kisker C, Kuper J, Van Houten B 2013 Prokaryotic nucleotide excision repair. Cold Spring Harb Perspect Biol 5:a012591. doi:10.1101/cshperspect.a012591. 23457260 PMC3578354

[22] Xie ZW, Liu SQ, Zhang YB, Wang ZG 2004 Roles of Rad23 protein in yeast nucleotide excision repair. Nucleic Acids Res 32:5981–5990. doi:10.1093/nar/gkh934. 15545636 PMC534619

[23] Ortolan TG, Chen L, Tongaonkar P, Madura K 2004 Rad23 stabilizes Rad4 from degradation by the Ub/proteasome pathway. Nucleic Acids Res 32:6490–6500. doi:10.1093/nar/gkh987. 15601997 PMC545455

[24] Zhou Z, Humphryes N, van Eijk P, Waters R, Yu S, Kraehenbuehl R, Hartsuiker E, Reed SH 2015 UV induced ubiquitination of the yeast Rad4-Rad23 complex promotes survival by regulating cellular dNTP pools. Nucleic Acids Res 43:7360–7370. doi:10.1093/nar/gkv680. 26150418 PMC4551923

[25] Lu DP, Christopher DA 2008 Endoplasmic reticulum stress activates the expression of a sub-group of protein disulfide isomerase genes and AtbZIP60 modulates the response in Arabidopsis thaliana. Mol Genet Genomics 280:199–210. doi:10.1007/s00438-008-0356-z. 18574595

[26] Chang M, Bellaoui M, Boone C, Brown GW 2002 A genome-wide screen for methyl methanesulfonate-sensitive mutants reveals genes required for S phase progression in the presence of DNA damage. Proc Natl Acad Sci U S A 99:16934–16939. doi:10.1073/pnas.262669299. 12482937 PMC139247

[27] Svensson JP, Pesudo LQ, Fry RC, Adeleye YA, Carmichael P, Samson LD 2011 Genomic phenotyping of the essential and non-essential yeast genome detects novel pathways for alkylation resistance. BMC Syst Biol 5:157. doi:10.1186/1752-0509-5-157. 21978764 PMC3213080

[28] Bailly V, Lauder S, Prakash S, Prakash L 1997 Yeast DNA repair proteins Rad6 and Rad18 form a heterodimer that has ubiquitin conjugating, DNA binding, and ATP hydrolytic activities. J Biol Chem 272:23360–23365. doi:10.1074/jbc.272.37.23360. 9287349

[29] Yan L, Xiong J, Lu H, Lv QZ, Ma QY, Cote P, Whiteway M, Jiang YY 2015 The role of Mms22p in DNA damage response in Candida albicans. G3 (Bethesda) 5:2567–2578. doi:10.1534/g3.115.021840. 26438292 PMC4683630

[30] Mukherjee PK, Zhou G, Munyon R, Ghannoum MA 2005 Candida biofilm: a well-designed protected environment. Med Mycol 43:191–208. doi:10.1080/13693780500107554. 16010846

[31] Norice CT, Smith FJ Jr, Solis N, Filler SG, Mitchell AP 2007 Requirement for Candida albicans Sun41 in biofilm formation and virulence. Eukaryot Cell 6:2046–2055. doi:10.1128/EC.00314-07. 17873081 PMC2168420

[32] Becker JM, Kauffman SJ, Hauser M, Huang L, Lin M, Sillaots S, Jiang B, Xu D, Roemer T 2010 Pathway analysis of Candida albicans survival and virulence determinants in a murine infection model. Proc Natl Acad Sci U S A 107:22044–22049. doi:10.1073/pnas.1009845107. 21135205 PMC3009777

[33] Braun BR, Head WS, Wang MX, Johnson AD 2000 Identification and characterization of TUP1-regulated genes in Candida albicans. Genetics 156:31–44. 10978273 10.1093/genetics/156.1.31 PMC1461230

[34] Jackson BE, Mitchell BM, Wilhelmus KR 2007 Corneal virulence of Candida albicans strains deficient in Tup1-regulated genes. Invest Ophthalmol Vis Sci 48:2535–2539. doi:10.1167/iovs.06-0909. 17525181

[35] Guzder SN, Sung P, Prakash L, Prakash S 1998 Affinity of yeast nucleotide excision repair factor 2, consisting of the Rad4 and Rad23 proteins, for ultraviolet damaged DNA. J Biol Chem 273:31541–31546. doi:10.1074/jbc.273.47.31541. 9813069

[36] Gao J, Wang H, Wong AH, Zeng G, Huang Z, Wang Y, Sang J, Wang Y 2014 Regulation of Rfa2 phosphorylation in response to genotoxic stress in Candida albicans. Mol Microbiol 94:141–155. doi:10.1111/mmi.12749. 25109320

[37] Verma S, Shakya VPS, Idnurm A 2019 The dual function gene RAD23 contributes to Cryptococcus neoformans virulence independently of its role in nucleotide excision DNA repair. Gene 717:144043. doi:10.1016/j.gene.2019.144043. 31400407

[38] Hiller E, Heine S, Brunner H, Rupp S 2007 Candida albicans Sun41p, a putative glycosidase, is involved in morphogenesis, cell wall biogenesis, and biofilm formation. Eukaryot Cell 6:2056–2065. doi:10.1128/EC.00285-07. 17905924 PMC2168408

[39] Firon A, Aubert S, Iraqui I, Guadagnini S, Goyard S, Prévost M-C, Janbon G, d'Enfert C 2007 The SUN41 and SUN42 genes are essential for cell separation in Candida albicans. Mol Microbiol 66:1256–1275. doi:10.1111/j.1365-2958.2007.06011.x. 18001349

[40] MacPherson S, Akache B, Weber S, De Deken X, Raymond M, Turcotte B 2005 Candida albicans zinc cluster protein Upc2p confers resistance to antifungal drugs and is an activator of ergosterol biosynthetic genes. Antimicrob Agents Chemother 49:1745–1752. doi:10.1128/AAC.49.5.1745-1752.2005. 15855491 PMC1087678

[41] Liu W, Zhao J, Li X, Li Y, Jiang L 2010 The protein kinase CaSch9p is required for the cell growth, filamentation and virulence in the human fungal pathogen Candida albicans. FEMS Yeast Res 10:462–470. doi:10.1111/j.1567-1364.2010.00617.x. 20345900

[42] Vyas VK, Barrasa MI, Fink GR 2015 A Candida albicans CRISPR system permits genetic engineering of essential genes and gene families. Sci Adv 1:e1500248. doi:10.1126/sciadv.1500248. 25977940 PMC4428347

[43] Murad AM, Lee PR, Broadbent ID, Barelle CJ, Brown AJ 2000 CIp10, an efficient and convenient integrating vector for Candida albicans. Yeast 16:325–327. 10669870 10.1002/1097-0061(20000315)16:4<325::AID-YEA538>3.0.CO;2-#

[44] Kumaran R, Yang SY, Leu JY 2013 Characterization of chromosome stability in diploid, polyploid and hybrid yeast cells. PLoS One 8:e68094. doi:10.1371/journal.pone.0068094. 23874507 PMC3707968

[45] Nobile CJ, Mitchell AP 2006 Genetics and genomics of Candida albicans biofilm formation. Cell Microbiol 8:1382–1391. doi:10.1111/j.1462-5822.2006.00761.x. 16848788

[46] Feng J, Islam A, Bean B, Feng J, Sparapani S, Shrivastava M, Goyal A, Omran RP, Mallick J, Whiteway M 15 1 2020 Hof1 plays a checkpoint related role in MMS induced DNA damage response in *Candida albicans*. Mol Biol Cell doi:10.1091/mbc.E19-06-0316. PMC7183792 31940254

