# Supplementary material for: Nucleotide Excision Repair Protein Rad23 Regulates Cell Virulence Independent of Rad4 in Candida albicans
Source: mSphere. 2020 Feb 19;5(1):e00062-20. doi: 10.1128/mSphere.00062-20 (PMC7031613; doi:10.1128/mSphere.00062-20)
Supplement: TABLE S1 [file mSphere.00062-20-st001.docx]

**Table S1 Strains used in this study**

| **Strains** | **Genotype** | **Source** |
| --- | --- | --- |
| SN148 | *arg4/arg4 leu2/leu2 his1/his1 ura3::imm434/ura3::imm434 iro1::imm434/iro1::imm43* | Noble S M, et al 2005 |
| JR7 | RM1000 *pph3::hisG/pph3::HIS1* | Feng J,et al 2013 |
| JC26 | SN148 *rad23::ARG4/rad23::ARG4* | This study |
| JJ1 | SN148 *rad23::ARG4/rad23::ARG4+CIP10* | This study |
| JJ2 | SN148 *rad23::ARG4/rad23::ARG4+CIP10-RAD23* | This study |
| JJ3 | SN148 *MET3p-RAD23::URA3/MET3p-RAD23::URA3* | This study |
| JJ4 | *JR7 rad23::ARG4/rad23::ARG4* | This study |
| JC19 | SN148 *rad4::ARG4/rad4::ARG4* | This study |
| JJ5 | SN148 *rad4::ARG4/rad4::ARG4 rad23::ARG4/rad23::ARG4* | This study |
| JC1 | *hof1::HIS1/hof1::HIS1* | (Manuscript in press) |
| JJ6 | *hof1::HIS1/hof1::HIS1 rad23::ARG4/rad23::ARG4* | This study |
| JJ7 | *rad53::ARG4/rad53::ARG4* | (Manuscript in press) |
| JJ8 | *rad23::ARG4/rad23::ARG4 rad53::ARG4/rad53::ARG4* | This study |
| JC17 | *mms22::ARG4/mms22::ARG4* | (Manuscript in press) |
| JJ9 | *rad23::ARG4/rad23::ARG4 mms22::ARG4/mms22::ARG4* | This study |
| JC21 | *rad18::ARG4/rad18::ARG4* | This study |
| JJ10 | *rad23::ARG4/rad23::ARG4 rad18::ARG4/rad18::ARG4* | This study |
| JC23 | *rad53::HIS1/rad53::HIS1* | This study |
| JJ11 | *rad53::HIS1/rad53::HIS1 hof1::ARG4/hof1::ARG4* | This study |
