# Supplementary material for: Nucleotide Excision Repair Protein Rad23 Regulates Cell Virulence Independent of Rad4 in Candida albicans
Source: mSphere. 2020 Feb 19;5(1):e00062-20. doi: 10.1128/mSphere.00062-20 (PMC7031613; doi:10.1128/mSphere.00062-20)
Supplement: TABLE S2 [file mSphere.00062-20-st002.docx]

**Table S2 primers used in this study**

| **Name** | **Oligos** | **Description** |
| --- | --- | --- |
| P7 | ATCTCATTAGATTTGGAACTTGTGGGTT | *CAS9* gene |
| P8 | TTCGAGCGTCCCAAAACCTTCT | *CAS9* gene |
| P1 | AAGAAAGAAAGAAAACCAGGAGTGAA | sgRNA |
| P4 | ACAAATATTTAAACTCGGGACCTGG | sgRNA |
| P5 | GCGGCCGCAAGTGATTAGACT | sgRNA |
| P6 | GCAGCTCAGTGATTAAGAGTAAAGATGG | sgRNA |
| RAD23-sg-F | AATGGATTAGTTTGGTTATCGTTTTAGAGCTAGAAATAGCAAGTTAAA | sgRNA for *RAD23* |
| RAD23-sg-R | GATAACCAAACTAATCCATTCAAATTAAAAATAGTTTACGCAAGTC | sgRNA for *RAD23* |
| RAD23-Re-F | ACGGCGAGAGGGCTGCTACGGGATGGCGCTATATTTGTACCACCGCCTCCCTTATTACCATCCAATACCTCGCCAGAAC | repair DNA for *RAD23* |
| RAD23-Re-R | TCATCGACACCTAAATCGGTGTTATTTTTAAGTGATAATATTAATGAAGTTAATGCTGCTCTCACTATAGGGAGACCG | repair DNA for *RAD23* |
| RAD23-Te-newF | TAGGGGTAGGTTCTGTATTG | genotype confirmation for *RAD23* |
| RAD23-Te-newR | TGACAACTACAGCCAAAACAG | genotype confirmation for *RAD23* |
| RAD23-F | CGGGGTACCGCCTTTAACACCACTTTGAG | amplification of *RAD23* gene |
| RAD23-R | CGGGGTACCATCTGGTTCAATAAAGGC | amplification of *RAD23* gene |
| sgRad23-promoter-F | TCAACCATAAGTTTAAGTGTGTTTTAGAGCTAGAAATAGCAAGTTAAA | sgRNA for *RAD23* promoter |
| sgRad23-promoter-R | ACACTTAAACTTATGGTTGACAAATTAAAAATAGTTTACGCAAGTC | sgRNA for *RAD23* promoter |
| Rad23-promoter-Re-F | TCTTTGTCTTCTAGCCATTGATTAATTTTTTTTTGGTTGCAAAGATTATAATAGTGGTTACGGATGGTATAAACGGAAAC | repair DNA for *RAD23* promoter |
| Rad23-promoter-Re-R | AGTCAATTCCACATCAAGTGACACGGTCTGTTTCTTGAAATCCTTGAATATAATTTGCATGTTTTCTGGGGAGGGTA | repair DNA for *RAD23* promoter |
| Rad23-promoter-Te-R | CACCCACTTCTCAATTCTG | genotype confirmation for *RAD23* promoter |
| SUN41-RT-F | GAATGGGGTGTCAAGAAAG | qRT-PCR |
| SUN41-RT-R | CCAAACACAACCATCTTCC | qRT-PCR |
| CEF3-RT-F | TCTTGCTGCTTTGGATGC | qRT-PCR |
| CEF3-RT-R | CTTTGACAGCAGTTGGGG | qRT-PCR |
| RBT-RT-F | CCACTGCTGAAATCGCCT | qRT-PCR |
| RBT4-RT-R | AGCATCGGCAGTGGGTTCA | qRT-PCR |
