# Supplementary material for: Nucleotide Excision Repair Protein Rad23 Regulates Cell Virulence Independent of Rad4 in Candida albicans
Source: mSphere. 2020 Feb 19;5(1):e00062-20. doi: 10.1128/mSphere.00062-20 (PMC7031613; doi:10.1128/mSphere.00062-20)
Supplement: TABLE S3 [file mSphere.00062-20-st003.docx]

**Table S3 Gene whose transcription was affected by deleting *RAD23***

| **Gene name** | **log2FC** | **Description** |
| --- | --- | --- |
| *orf19.4922* | 1.45 | Ortholog(s) have Rab GTPase binding activity and Golgi apparatus localization |
| *orf19.4474* | 1.25 | Ortholog(s) have proteasome binding activity and role in cellular response to arsenic-containing substance, proteasome-mediated ubiquitin-dependent protein catabolic process |
| *orf19.2472* | 1.25 | Ortholog of S. pombe replication termination factor Rtf2; Spider biofilm induced |
| *ZPR1* | 1.18 | Protein with putative zinc finger; regulated by Gcn4p; repressed in response to amino acid starvation (3-aminotriazole treatment); upregulation correlates with clinical development of fluconazole resistance |
| *ARG3* | 1.15 | Putative ornithine carbamoyltransferase; Gcn4-regulated; Hap43-induced; repressed in alkalinizing medium; rat catheter and Spider biofilm induced |
| *PCL5* | 1.11 | Putative cyclin for Pho85 kinase; Gcn4-induced; suppresses toxicity of C. albicans Gcn4 overproduction in S. cerevisiae via increased Pho85-dependent phosphorylation and degradation of Gcn4; rat catheter and Spider biofilm induced |
| *LEU42* | 1.10 | Putative alpha-isopropylmalate synthase; fungal-specific; induced by human blood or polymorphonuclear cells; regulated by Gcn2 and Gcn4; stationary phase enriched protein; Spider biofilm induced |
| *orf19.5504* | 1.09 | Ortholog of C. dubliniensis CD36 : Cd36_73400, C. parapsilosis CDC317 : CPAR2_703180, Candida tenuis NRRL Y-1498 : CANTEDRAFT_104803 and Debaryomyces hansenii CBS767 : DEHA2A04466g |
| *orf19.1267.1* | 1.07 | Ortholog(s) have cysteine desulfurase activity, role in iron-sulfur cluster assembly and L-cysteine desulfurase complex, extrinsic component of mitochondrial inner membrane, mitochondrial matrix localization |
| *orf19.5235* | 1.06 | Putative mitochondrial ribosomal protein of the large subunit; Hap43-induced; mutants are viable; protein level decreases in stationary phase |
| *orf19.35.1* | 0.98 | Protein of unknown function |
| *BUL1* | 0.96 | Protein similar but not orthologous to S. cerevisiae Bul1; a protein involved in selection of substrates for ubiquitination; mutants are viable; macrophage/pseudohyphal-induced; rat catheter biofilm induced |
| *orf19.3351* | 0.90 | Protein of unknown function; Hap43-induced; Spider biofilm induced |
| *orf19.1394* | 0.90 | Putative protein of unknown function; mutant is viable; protein level decreases in stationary phase cultures; Spider biofilm induced |
| *NIP7* | 0.87 | Putative nucleolar protein with role in ribosomal assembly; hyphal-induced; Hap43-induced; Spider biofilm induced |
| *orf19.5619* | 0.86 | Protein of unknown function; induced by alpha pheromone in SpiderM medium; Spider biofilm induced |
| *HIS3* | 0.84 | Imidazoleglycerol-phosphate dehydratase, enzyme of histidine biosynthesis; functionally complements S. cerevisiae his3-1 mutation; hyphal-induced expression; regulated by Gcn2p and Gcn4p; fungal-specific (no human or murine homolog) |
| *IMG2* | 0.84 | Mitochondrial ribosomal protein of the large subunit; rat catheter biofilm induced |
| *PEX17* | 0.81 | Putative peroxin |
| *orf19.1406* | 0.80 | Ortholog(s) have DNA-directed DNA polymerase activity, role in error-free translesion synthesis, error-prone translesion synthesis and mitochondrion, nuclear chromatin, zeta DNA polymerase complex localization |
| *orf19.1993* | 0.80 | Ortholog(s) have structural molecule activity, role in proteasome assembly, ubiquitin-dependent protein catabolic process and cytosol, nucleus, proteasome regulatory particle, lid subcomplex, proteasome storage granule localization |
| *orf19.2191.1* | 0.75 | Ortholog of C. dubliniensis CD36 : Cd36_11730, C. parapsilosis CDC317 : CPAR2_104195, Candida tenuis NRRL Y-1498 : CANTEDRAFT_109454 and Debaryomyces hansenii CBS767 : DEHA2C10076g |
| *ARC18* | 0.73 | Putative ARP2/3 complex subunit; mutation confers hypersensitivity to cytochalasin D |
| *TFB3* | 0.71 | Putative C3HC4 zinc finger transcription factor; transcript positively regulated by Tbf1; Spider biofilm induced |
| *LYS14* | 0.69 | Zn(II)2Cys6 transcription factor; has similarity to S. cerevisiae Lys14, which is a transcription factor involved in the regulation of lysine biosynthesis genes |
| *LYS22* | 0.68 | Homocitrate synthase, minor isoform; repressed by nitric oxide and by hypoxia; protein level decreases in stationary phase cultures; induced by ketoconazole, Spider biofilm induced; flow model biofilm repressed |
| *CRD2* | 0.67 | Metallothionein; for adaptation to growth in high copper; basal transcription is cadmium-repressed; Ssn6 regulated; complements copper sensitivity of an S. cerevisiae cup1 mutant; regulated by Sef1, Sfu1, and Hap43; Spider biofilm induced |
| *RPN6* | 0.67 | Putative 26S proteasome subunit; Hap43p-induced gene; regulated by Gcn2p and Gcn4p |
| *TOA2* | 0.67 | Putative TFIIA small subunit; protein abundance decreased in CAI4 strain compared to the SC5314 strain, abundance not affected by reintegration of URA3 in CAI4; flucytosine induced; possibly an essential gene (UAU1 method) |
| *orf19.1707* | 0.66 | Ortholog of *Candida albicans WO-1* : CAWG_02476 |
| *orf19.5628* | 0.66 | Mitochondrial dicarboxylate transporter; possibly an essential gene, disruptants not obtained by UAU1 method |
| *TAP42* | 0.65 | Ortholog(s) have role in TOR signaling, positive regulation of transcription by RNA polymerase I and cytosol, extrinsic component of membrane localization |
| *orf19.1340* | 0.64 | Putative aldose reductase; protein level decreases in stationary phase cultures; Spider biofilm repressed |
| *orf19.6340* | 0.63 | Ortholog(s) have DNA-directed 5'-3' RNA polymerase activity, RNA polymerase I activity, RNA polymerase II activity, RNA polymerase III activity, RNA-directed 5'-3' RNA polymerase activity |
| *NOC2* | 0.62 | Putative nucleolar complex protein; Hap43-induced; transposon mutation affects filamentous growth; mutation confers hypersensitivity to 5-fluorouracil (5-FU), tubercidin (7-deazaadenosine); repressed in core stress response |
| *YIM1* | 0.62 | Protein similar to protease of mitochondrial inner membrane; increased transcription is observed upon benomyl treatment; macrophage-downregulated gene |
| *ACP1* | 0.61 | Putative mitochondrial acyl carrier protein involved in fatty acid biosynthesis; shows colony morphology-related gene regulation by Ssn6p; protein newly produced during adaptation to the serum |
| *PRN4* | 0.60 | Protein with similarity to pirins; induced by benomyl treatment; flow model biofilm repressed |
| *GIM5* | 0.59 | Putative heterohexameric cochaperone prefoldin complex subunit; macrophage/pseudohyphal-repressed gene and macrophage-induced protein |
| [*orf19.1075.1*](http://www.candidagenome.org/cgi-bin/locus.pl?locus=orf19.1075.1&seq_source=C.%20albicans%20SC5314%20Assembly%2021) | 0.58 | Pseudogene; added to Assembly 21 based on comparative genome analysis |
| [*orf19.1815*](http://www.candidagenome.org/cgi-bin/locus.pl?locus=orf19.1815&seq_source=C.%20albicans%20SC5314%20Assembly%2021) | 0.56 | Ortholog of S. cerevisae/S. pombe Tif6; constituent of 66S pre-ribosomal particles; Spider biofilm induced |
| *TRP3* | 0.55 | Putative bifunctional enzyme with predicted indole-3-glycerol-phosphate synthase and anthranilate synthase activities; regulated by Gcn2p and Gcn4p |
| [*orf19.1516*](http://www.candidagenome.org/cgi-bin/locus.pl?locus=orf19.1516&seq_source=C.%20albicans%20SC5314%20Assembly%2021) | 0.53 | Ortholog(s) have thiol-dependent ubiquitin-specific protease activity and role in negative regulation of gluconeogenesis, proteasome-mediated ubiquitin-dependent protein catabolic process |
| *DAO2* | 0.52 | Putative D-amino acid oxidase; rat catheter biofilm induced |
| *ILV6* | 0.51 | Putative regulatory subunit of acetolacetate synthase; alkaline induced; regulated by Gcn2 and Gcn4; protein present in exponential and stationary growth phase yeast; Spider biofilm repressed |
| *HOM6* | 0.51 | Putative homoserine dehydrogenase; Gcn4-regulated; induced by amino acid starvation (3-ATtreatment); macrophage-induced protein; protein level decreases in stationary phase cultures; flow model biofilm repressed |
| [*orf19.1246*](http://www.candidagenome.org/cgi-bin/locus.pl?locus=orf19.1246&seq_source=C.%20albicans%20SC5314%20Assembly%2021) | -0.50 | Putative eisosome component role in proper eisosome assembly; upregulated in cyr1 null mutant |
| *TOS4* | -0.50 | Putative fork-head transcription factor; rat catheter and Spider biofilm repressed |
| [*orf19.1514*](http://www.candidagenome.org/cgi-bin/locus.pl?locus=orf19.1514&seq_source=C.%20albicans%20SC5314%20Assembly%2021) | -0.52 | Ortholog(s) have enzyme binding, phosphatidylinositol-4-phosphate binding activity |
| *HSL1* | -0.52 | Probable protein kinase involved in determination of morphology during the cell cycle of both yeast-form and hyphal cells via regulation of Swe1p and Cdc28p; required for full virulence and kidney colonization in mouse systemic infection |
| [*orf19.3080*](http://www.candidagenome.org/cgi-bin/locus.pl?locus=orf19.3080&seq_source=C.%20albicans%20SC5314%20Assembly%2021) | -0.54 | Ortholog(s) have double-stranded DNA binding, kinetochore binding activity |
| [*orf19.5274*](http://www.candidagenome.org/cgi-bin/locus.pl?locus=orf19.5274&seq_source=C.%20albicans%20SC5314%20Assembly%2021) | -0.54 | Ortholog of *S. cerevisiae* : SKG3, *C. dubliniensis CD36* : Cd36_11180, *C. parapsilosis CDC317* : CPAR2_206250, *C. auris B8441* : B9J08_005263 and *Debaryomyces hansenii CBS767* : DEHA2E13794g |
| [*orf19.3003*](http://www.candidagenome.org/cgi-bin/locus.pl?locus=orf19.3003&seq_source=C.%20albicans%20SC5314%20Assembly%2021) | -0.54 | Putative lipid-binding protein with a predicted role in calcium-dependent phospholipid-binding |
| [*orf19.1409.1*](http://www.candidagenome.org/cgi-bin/locus.pl?locus=orf19.1409.1&seq_source=C.%20albicans%20SC5314%20Assembly%2021) | -0.55 | Ribosomal 60S subunit protein L22B; Spider biofilm repressed |
| [*orf19.5555*](http://www.candidagenome.org/cgi-bin/locus.pl?locus=orf19.5555&seq_source=C.%20albicans%20SC5314%20Assembly%2021) | -0.55 | Ortholog of *C. dubliniensis CD36* : Cd36_63300, *Candida tropicalis NEW ASSEMBLY* : CTRG1_05769, *Candida tropicalis MYA-3404* : CTRG_05769 and *Candida albicans WO-1* : CAWG_05077 |
| *GPM1* | -0.56 | Phosphoglycerate mutase; surface protein that binds host complement Factor H and FHL-1; antigenic; fluconazole, or amino acid starvation (3-AT) induced, farnesol-repressed; Hap43, flow model biofilm induced; Spider biofilm repressed |
| [*orf19.4043*](http://www.candidagenome.org/cgi-bin/locus.pl?locus=orf19.4043&seq_source=C.%20albicans%20SC5314%20Assembly%2021) | -0.58 | Protein with a predicted pleckstrin homology domain; induced by alpha pheromone in SpiderM medium |
| *HTA1* | -0.58 | Histone H2A; repressed in fkh2 mutant; amphotericin B repressed; farnesol regulated; RNA abundance regulated by tyrosol and cell density; Hap43-induced gene; Spider biofilm repressed |
| *MP65* | -0.59 | Cell surface mannoprotein; cell-wall glucan metabolism, adhesion; adhesin motif; O-glycosylation; induced by heat, germ tube formation, wall regeneration; mycelial antigen; diagnostic marker; fluconazole-repressed; Spider biofilm induced |
| *ERG3* | -0.59 | C-5 sterol desaturase; introduces C-5(6) double bond into episterol; some clinical isolates show increased azole resistance and defects in hyphal growth and virulence; Efg1p-repressed; fluconazole-induced |
| *PTR22* | -0.60 | Oligopeptide transporter involved in uptake of di-/tripeptides; regulated by Stp2 and Stp3; transcript induced upon phagocytosis by macrophage; repressed by Rim101 at pH 8; flow model biofilm induced |
| *OLE1* | -0.60 | Fatty acid desaturase, essential protein involved in oleic acid synthesis; required for aerobic hyphal growth and chlamydospore formation; subject to hypoxic regulation; fluconazole-induced; caspofungin repressed; Hap43p-induced |
| *BMT4* | -0.65 | Beta-mannosyltransferase; for elongation of beta-mannose chains on the acid-labile fraction of cell wall phosphopeptidomannan; 9-gene family member; regulated by Tsa1, Tsa1B; flow model biofilm induced; rat catheter biofilm repressed |
| *SWE1* | -0.68 | Putative protein kinase with a role in control of growth and morphogenesis, required for full virulence; mutant cells are small, rounded, and sometimes binucleate; not required for filamentous growth; mutant is hypersensitive to caspofungin |
| *RPS26A* | -0.69 | Ribosomal protein; regulated by Nrg1, Tup1; repressed upon phagocytosis by murine macrophage; alternatively spliced intron in 5'-UTR; Spider biofilm repressed |
| *EFG1* | -0.69 | bHLH transcription factor; required for white-phase cell type, RPMI and Spider biofilm formation, hyphal growth, cell-wall gene regulation; roles in adhesion, virulence; Cph1 and Efg1 have role in host cytokine response; binds E-box |
| *PGA54* | -0.71 | GPI-anchored protein; Hog1-repressed; induced in cyr1 or efg1 mutant or in hyphae; colony morphology-related gene regulation by Ssn6; induced in RHE model; mRNA binds She3; regulated in Spider biofilms by Tec1, Egf1, Ntd80, Rob1, Brg1 |
| *IST2* | -0.75 | Ortholog(s) have lipid binding activity and role in endoplasmic reticulum membrane organization, protein localization to plasma membrane, regulation of phosphatidylinositol dephosphorylation |
| *GIT3* | -0.76 | Glycerophosphocholine permease; white cell specific transcript; fungal-specific; alkaline repressed; caspofungin, macrophage/pseudohyphal-repressed; flow model biofilm induced; Spider biofilm induced |
| *orf19.6874* | -0.76 | Putative helix-loop-helix (HLH) transcription factor with a role in filamentous growth |
| *orf19.2312* | -0.77 | Protein similar to ferric reductase Fre10p; possibly an essential gene, disruptants not obtained by UAU1 method |
| *orf19.4657* | -0.79 | Ortholog(s) have phosphoprotein phosphatase activity and role in negative regulation of phospholipid biosynthetic process, nuclear envelope organization, positive regulation of phosphatidate phosphatase activity, protein dephosphorylation |
| *orf19.3051* | -0.79 | Protein of unknown function; S. pombe ortholog SPAC17A2.02c plays a role in resistance to cadmium; colony morphology-related gene regulation by Ssn6; Spider biofilm repressed |
| *CWH8* | -0.80 | 1.5 |
| *orf19.323* | -0.81 | Putative haloacid dehalogenase; localized to plasma membrane |
| *orf19.1495* | -0.81 | Ortholog(s) have role in L-methionine salvage from methylthioadenosine and cytosol localization |
| *PMA1* | -0.83 | Plasma membrane H(+)-ATPase; highly expressed, comprises 20-40% of total plasma membrane protein; levels increase at stationary phase transition; fluconazole induced; caspofungin repressed; upregulated in RHE model; Spider biofilm repressed |
| *RBT4* | -0.87 | Pry family protein; required for virulence in mouse systemic/rabbit corneal infections; not filamentation; mRNA binds She3, is localized to hyphal tips; Hap43-induced; in both yeast and hyphal culture supernatants; Spider biofilm induced |
| *orf19.2296* | -0.95 | Predicted mucin-like protein; ketoconazole-induced; fluconazole-repressed; induced in cyr1 mutant; colony morphology-related gene regulation by Ssn6; flow model biofilm induced; Spider biofilm induced |
| *ALS9* | -1.02 | ALS family cell-surface glycoprotein; expressed during infection of human epithelial cells; confers laminin adhesion to S. cerevisiae; highly variable; putative GPI-anchor; Hap43-repressed |
| *MTD1* | -1.04 | Ortholog(s) have methylenetetrahydrofolate dehydrogenase (NAD+) activity, role in folic acid-containing compound biosynthetic process, one-carbon metabolic process, purine nucleobase biosynthetic process and cytosol localization (5) |
| *ALS4* | -1.18 | GPI-anchored adhesin; role in adhesion, germ tube induction; growth, temperature regulated; expressed during infection of human buccal epithelial cells; repressed by vaginal contact; biofilm induced; repressed during chlamydospore formation |
| *orf19.1691* | -1.42 | Plasma-membrane-localized protein; filament induced; Hog1, ketoconazole, fluconazole and hypoxia-induced; regulated by Nrg1, Tup1, Upc2; induced by prostaglandins; flow model biofilm induced; rat catheter and Spider biofilm repressed |
| *FGR23* | -2.15 | Protein of unknown function; repressed by a1/alpha2 in white-phase cells, a-specific, alpha factor-induced; Hap43-repressed; flow model biofilm induced; Tn mutation affects filamentous growth |
| *CEF3* | -2.35 | Translation elongation factor 3; antigenic in humans; predicted C-term nucleotide-binding active site; protein on surface of yeast, not hyphae; polystyrene adherence induced; higher protein amount in stationary phase; possibly essential |
| *SUN41* | -2.43 | Cell wall glycosidase; role in biofilm formation and cell separation; possibly secreted; hypoxia, hyphal induced; caspofungin repressed; Efg1, Cph1 regulated; O-glycosylated, possible Kex2 substrate; 5'-UTR intron; Spider biofilm induced |
| *Rad23* | -7.93 | S. cerevisiae Rad23 ortholog; binds damaged DNA; Spider biofilm repressed |
